# Interface Analysis of MOCVD Grown GeTe/Sb_2_Te_3_ and Ge-Rich Ge-Sb-Te/Sb_2_Te_3_ Core-Shell Nanowires

**DOI:** 10.3390/nano12101623

**Published:** 2022-05-10

**Authors:** Arun Kumar, Seyed Ariana Mirshokraee, Alessio Lamperti, Matteo Cantoni, Massimo Longo, Claudia Wiemer

**Affiliations:** 1 CNR—Institute for Microelectronics and Microsystems, Via C. Olivetti 2, 20864 Agrate Brianza, Italy; s.mirshokraee@campus.unimib.it (S.A.M.); alessio.lamperti@mdm.imm.cnr.it (A.L.); claudia.wiemer@mdm.imm.cnr.it (C.W.); 2 Department of Physics ‘E.R. Caianiello’, University of Salerno, Via G. Paollo I 132, 84084 Salerno, Italy; 3 Department of Physics, Politecnico di Milano, Via G. Colombo 81, 20133 Milano, Italy; matteo.cantoni@polimi.it; 4 CNR—Institute for Microelectronics and Microsystems, Via del Fosso del Cavaliere 100, 00133 Rome, Italy

**Keywords:** MOCVD, XPS, Ge-rich Ge-Sb-Te/Sb_2_Te_3_, GeTe/Sb_2_Te_3_, core-shell nanowires

## Abstract

Controlling material thickness and element interdiffusion at the interface is crucial for many applications of core-shell nanowires. Herein, we report the thickness-controlled and conformal growth of a Sb_2_Te_3_ shell over GeTe and Ge-rich Ge-Sb-Te core nanowires synthesized via metal-organic chemical vapor deposition (MOCVD), catalyzed by the Vapor–Liquid–Solid (VLS) mechanism. The thickness of the Sb_2_Te_3_ shell could be adjusted by controlling the growth time without altering the nanowire morphology. Scanning electron microscopy (SEM) and X-ray diffraction (XRD) techniques were employed to examine the surface morphology and the structure of the nanowires. The study aims to investigate the interdiffusion, intactness, as well as the oxidation state of the core-shell nanowires. Angle-resolved X-ray photoelectron spectroscopy (XPS) was applied to investigate the surface chemistry of the nanowires. No elemental interdiffusion between the GeTe, Ge-rich Ge-Sb-Te cores, and Sb_2_Te_3_ shell of the nanowires was revealed. Chemical bonding between the core and the shell was observed.

## 1. Introduction

Interest in Phase Change Memories (PCMs) based on chalcogenide alloys continues to grow due to the wide range of possible applications that can be reached by the reversible amorphous–to–crystalline phase transition of chalcogenide materials [1,2,3,4,5,6]. PCMs are the most promising candidate for realizing “Storage Class Memories”, which could fill the gap between ‘‘operation’’ and ‘‘storage’’ memories [7,8]. The main improvements needed to exploit the full potential of PCMs in these innovative applications are the reduction of the programming currents and further cell downscaling. In particular, the reduction in the programming currents is related to lower energy to induce the phase transitions, hence lower power consumption. However, other limitations, such as alloy composition, structure, small cell size, and high scalability, exist in the path of the realization of PCMs [9]. Further, multilevel PCMs’ heterostructures have been explored by properly pairing two or more phase change materials featuring different crystallization properties. Compared to the commonly employed Ge-Sb-Te (GST) and GeTe (GT) alloys in PCM cells, Sb_2_Te_3_ (ST) exhibits a lower crystallization temperature and faster reversible switching, making it an ideal candidate for the realization of heterostructure-based multi-level PCM cells, in which the different properties of the involved materials can be combined to improve the overall performances, and for carrying out fundamental studies on the role of the heterointerface in the resistive switch.

Among the different methods explored to overcome the above limitations, nanowire (NWs) based PCMs fabricated by the bottom-up approach have drawn considerable interest [10,11,12,13,14,15]. Advantages of using NWs for such an application are their small sub lithographic feature sizes and single-crystalline defect-free structure, where novel functionalities are expected to originate by engineering the constituent compositions, sizes, and structures, as in the case of axial [16,17,18], radial (core-shell) [19,20], and branched heterostructured NWs [21]. Various PCM cells based on the GT and GST structures have been previously reported [22,23,24,25,26,27,28]. Core-shell NWs formed by two chalcogenide materials with different phase change characteristics, namely Ge_2_Sb_2_Te_5_/GeTe, have also been proposed as multi-level PCM memory cells [29]. Indeed, the growth and the properties of Ge-rich Ge-Sb-Te/Sb_2_Te_3_ (GGST/ST) and GeTe/Sb_2_Te_3_ (GT/ST) PCM core-shell NWs have been recently reported by our group [30,31].

In the present work, we examined the morphological and structural characteristics of the GT/ST and GGST/ST core-shell NWs via Field Emission Scanning electron microscopy (FESEM) and X-ray diffraction (XRD), respectively. A detailed investigation was carried out on the elemental composition and chemical bonding of the MOCVD-grown NWs by exploiting X-ray photoelectron spectroscopy (XPS) analysis on both core and core-shell structures, to extract information on the existing nanointerfaces between the different core and shell materials.

## 2. Experimental Section

The growth of the GGST and GT core NWs was carried out with an Aixtron AIX200/4 MOCVD reactor (Aixtron SE, Herzogenrath, Germany), employing the Vapor–Liquid–Solid (VLS) mechanism catalyzed by Au nanoparticles (NPs), with average sizes of 20, 30, and 50 nm. Details about the growth methodology have been previously reported [30,31]. The GT core NWs were obtained with optimized reactor temperature (T), reactor pressure (P), and a growth duration (t) of 400 °C, 50 mbar, and 60 min, respectively. The required precursor pressure for the GT growth was 3.35 × 10^−3^ mbar for tetraisdimethylamino germanium (Ge[N(CH_3_)_2_]_4_, TDMAGe), and 8.58 × 10^−3^ mbar for diisopropyl telluride ((C_3_H_7_)_2_Te, DiPTe). The GGST core NWs were obtained with the optimized T, P, and t parameters of 400 °C, 50 mbar, and 60 min, respectively. The required precursor pressures were 4.42 × 10^−3^ mbar for tetraisdimethylamino germanium (Ge[N(CH_3_)_2_]_4_, TDMAGe), 5.12 × 10^−5^ mbar for antimony trichloride (SbCl_3_) and 6.98 × 10^−3^ mbar for diisopropyl telluride ((C_3_H_7_)_2_Te, DiPTe. The chemical composition of the GGST NWs has been previously estimated by electron energy loss spectroscopy (EELS) to be 35% Ge, 10% Sb, 55% Te, corresponding to the atomic ratio of Ge:Sb:Te = 3:1:5, with the Ge concentration higher than regular Ge_2_Sb_2_Te_5_ [30].

The ST shell deposition on the core NWs was performed at room temperature, by employing the SbCl_3_ and bis(trimethylsilyl) telluride (Te(SiMe_3_)_2_, DSMTe) precursors, with the partial pressures of 2.23 × 10^−4^ mbar and 3.25 × 10^−4^ mbar, respectively. The growth rate of ST was optimized to be 20 nm/h.

The surface morphology in-plane and cross-section mode of the obtained NWs was carried out using a ZeissR© Supra40 field-emission scanning electron microscope (FE-SEM) (Cark Zeiss, Oberkochen, Germany). XRD analysis was performed using an Ital Structures HRD3000 diffractometer system (Ital Structures Sas, Riva de Garda, Italy) to evaluate the average crystal structure of the obtained NWs. The experimental XRD patterns were analyzed by the MAUD program. XPS was performed by an XPS ESCA 5600 apparatus (monochromatic Al K_α_ X-ray source, 1486.6 eV) equipped with a concentric hemispherical analyzer (Physical Electronics Inc., Chanhassen, MN, USA) to investigate the elemental composition and the chemical bonding of NWs, and the interface between the core and shell of the core-shell NWs. Pass energy was 58.50 eV with energy steps of 0.25 eV. Binding energies were calibrated considering the C_1s peak at 285 eV. All the related XPS measurements were analyzed and fitted by the XPSPEAK41 program, using a Voigt shape for each experimental peak.

## 3. Results and Discussion

**Figure 1:**
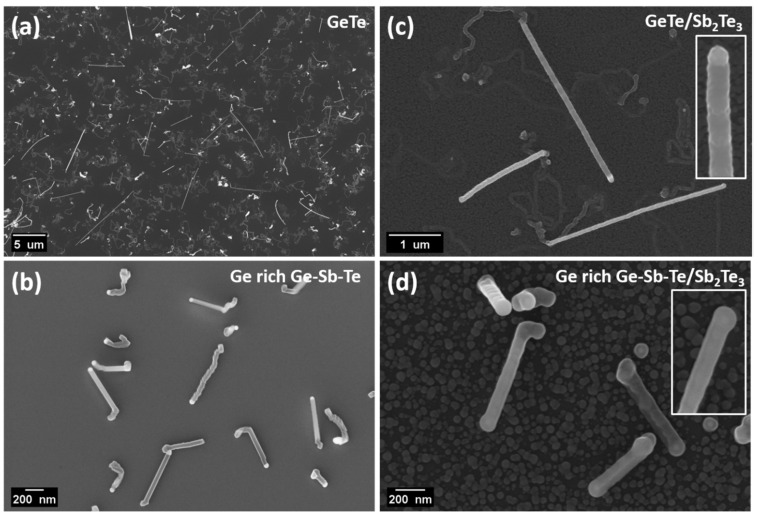
Top-view SEM image of (**a**) GT, (**b**) GGST core; and (**c**) GT/ST, (**d**) GGST/ST core-shell NWs with 10 nm shell thickness, insets show the magnified view of a single NW.

Figure 1a,b shows the top-view SEM images of the GT and GGST core NWs grown on Si and SiO_2_ substrates, respectively. It is useful to recall here the results of the morphological analysis from our previous works [30,31], Au catalyst NPs were observed at the NWs’ tips, confirming that the growth occurs via the VLS mechanism; the as-grown GT NWs were of an average length and diameter of about 5 µm and 50 nm, while the GGST NWs were about 1.40 µm long and 60 nm in diameter, respectively; the diameter of the NWs turned out to be directly dependent on the size of the catalyzed Au NPs [30,31]. Figure 1c,d depicts the GT/ST and GGST/ST core-shell NWs having shells of about 10 nm thickness, and the insets show the magnified view of a single NW. The 10 nm shell revealed a lower granularity in comparison to the 30 nm shell [30,31]. The shell was continuously deposited all over the core NWs. Such nanostructures with 10 nm and 30 nm shell thicknesses (obtained for a deposition time of 30 and 90 min, respectively) were, therefore, the subject of the present study, with a special focus on the core-shell interface.

**Figure 2:**
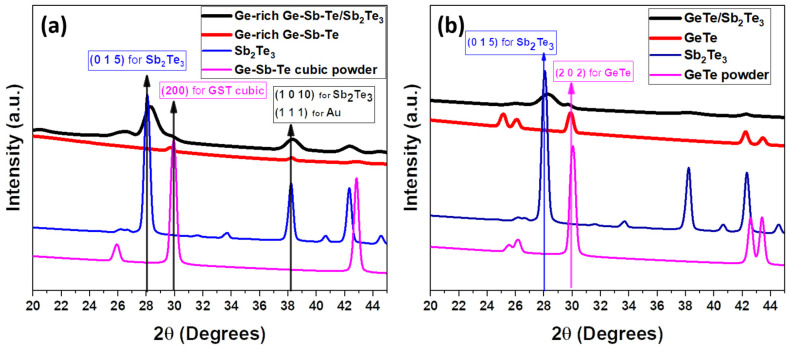
XRD patterns of (**a**) GGST, and GGST/ST core-shell NWs; (**b**) GT, and GT/ST core-shell NWs.

Figure 2 shows the XRD patterns obtained from the core and core-shell NWs. The patterns were simulated by taking into account not only the peak position but also the existing background and peak broadening, using the open-source software Maud [32]. Figure 2a shows a set of XRD patterns obtained for the GGST core and GGST/ST core-shell NWs. The GGST core NWs exhibited broad diffraction peaks centered at the 2θ values expected for the face-centered cubic Ge_2_Sb_2_Te_5_ phase, along with the presence of the cubic Au NPs diffraction peak at about 38.1° (Figure 2a). Thus, the crystallized cubic GGST, exhibits the lattice parameters of cubic Ge_2_Sb_2_Te_5_ [30]. In the case of GGST/ST core-shell NWs, the Ge_2_Sb_2_Te_5_ peak is revealed as a shoulder close to the (015) reflection of ST, while the diffraction peaks from the Sb_2_Te_3_ phase are clearly visible (Figure 2a). Further, Figure 2b shows the XRD patterns of the GT and GT/ST core-shell NWs. The GT NWs were found to exhibit rhombohedral structure. The extracted lattice parameters of the NWs were found to be a = 8.28 Å, and c = 10.55 Å. In the pattern of the GT/ST core-shell NWs in Figure 2b, there is a shoulder at the right side of the (015) reflection of ST, at around 2θ = 29.6°, that could be attributed to the (202) main reflection of the GT structure, confirming that the core GT NWs preserve their crystallinity after the ST deposition. The extracted lattice parameters of Sb_2_Te_3_ by Rietveld refinements were found to be a = 4.22 Å and c = 30.46 Å [31]. This also confirms that no structural disordering occurred with the deposited shell.

XPS analysis was employed to investigate the elemental composition and chemical bonding of the NWs in the form of core and core-shell nanostructures. In order to study the interfacial interaction between the core NWs and their corresponding shells, the XPS analysis of core-shell NWs was compared to the same analysis over the corresponding NWs without their shells. A detailed list of the samples analyzed and their related experimental XPS peak positions and FWHM are reported in the Supplementary Materials (Table S1).

The ex-situ XPS characterizations were performed straightaway after the NWs growth, to prevent the oxidation of the samples. The measurements were recorded with different take-off angles, i.e., the angle between the surface and detector. When samples are probed with a larger take-off angle, up to 90 degrees, the probed thickness through them is larger. By decreasing the take-off angle, shallower volumes are probed. Thus, upon changing the take-off angle, different volumes below the sample surface can be investigated, and this will be particularly relevant when different shell thicknesses are investigated.

If the relative intensities of the probed signals change by varying the take-off angles, we can predict that the signals are generated from our investigated material (NWs and Sb_2_Te_3_ thin film over the surface, as well as on the NWs) and not from the environmental contamination (such as carbon). The NWs grew uniformly over the surface, although along random orientations; most of them grew horizontally parallel to the surface, thus are suitable for XPS investigation. Exploiting the angle dependence of the measured depth, when no signal from the Ge atoms was detected, that were not expected in the shell, we were sure in particular to measure in the shell only.

**Figure 3:**
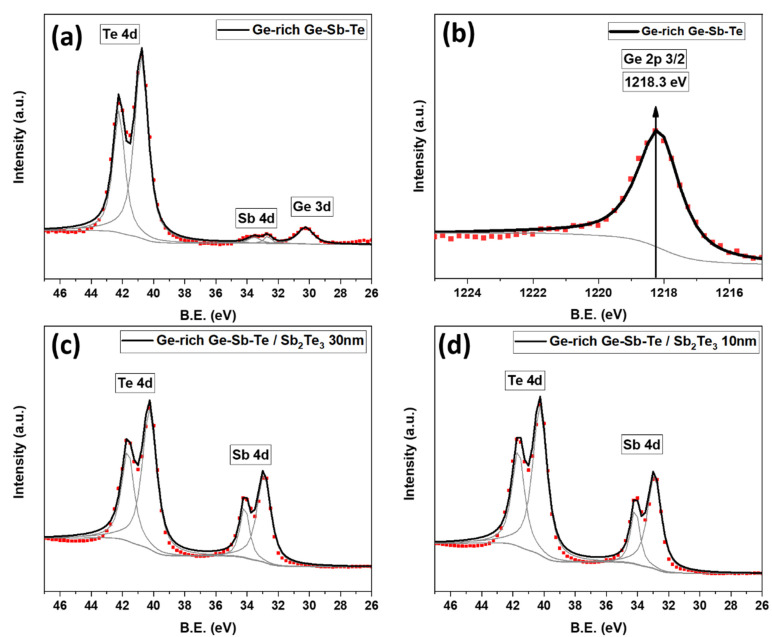
XPS spectra for (**a**) Te_4d, Sb_4d; and (**b**) Ge_2p region for GGST core NWs; (**c**) Te_4d, Sb_4d region for GGST/ST core-shell NWs with 30 nm shell thickness; (**d**) Te_4d, Sb_4d region for GGST/ST core-shell NWs with 10nm shell thickness; (dots–experimental data; solid lines—fit).

**Figure 4:**
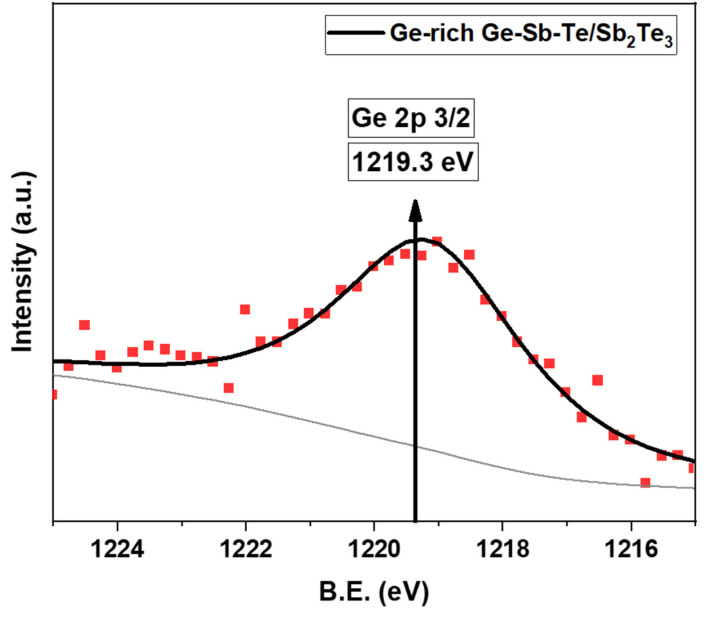
XPS spectra for Ge_2p_3/2_ orbital of GGST/ST core-shell NWs with 10 nm shell thickness; (dots–experimental data; solid lines—fit).

As a first step, the sample containing GGST NWs catalyzed with Au NPs size of 50 nm size, was examined. The dots in the figures are the raw data, and the solid lines are the fitted data. In addition, in some figures, there are some lines of a gray color that show the individual deconvoluted peaks used for fitting. Figure 3a shows the XPS spectra of Ge, Sb, and Te obtained by analyses of the Ge_3d, Sb_4d, and Te_4d core level peaks. The gray color shows the inelastic background, fitted by the Shirley method, and the individual deconvoluted peaks used for fitting. The same data representation and analysis apply to Figure 4. Figure 3b shows the XPS spectrum of the Ge_2p core level, which has higher binding energy (1218 eV), and thus a smaller mean free path.

The inelastic mean free path (IMFP) is an index of how far an electron travels on average through a solid before losing energy, and can be calculated by the following formula:

I_(d)_ = I_0_ e^(−d/λ(E))^ (1)

where I_(d)_ is the intensity after the primary electron beam has traveled through the solid to a distance d. I_0_ is the intensity of the primary electrons, and λ is the interaction mean free path [33].

By considering IMFP for Ge_2p orbital electrons, which is about 0.9 nm, we can exclude that these electrons can probe an under layer buried by a 10 nm thick layer. On the contrary, if the shell thickness was not uniform, a Ge signal should emerge from the core, provided that, in some parts, the thickness is definitely thinner than 10 nm, comparable with the IMFP.

In the Ge_2p range of energy, except Ge_2p, none of the NW shell (Te and Sb) XPS peaks present. So, we could study only the Ge signal from the NW core without any disturbance from the Sb_2_Te_3_ contributions, that could overlap with the peak, making the analysis more difficult and noisier. Ge is important for core and core-shell interface probing, because it is the only element present in the core of NWs but not in their shell. Thus, the Ge signal can give us information about the bulk and the interface of the NWs. The measurements confirmed the stability of Sb and Te [34]. The GGST experimental peak positions were clearly identified for each element and located at 30.20 ± 0.1 eV (Ge_3d), 1218.3 ± 0.1 eV (Ge_2p_3/2_), 32.70 ± 0.1 eV (Sb_4d), and 40.15 ± 0.1  eV (Te_4d). The obtained spectrum confirmed the absence of oxidation on the NWs, as no extra peaks or shoulder tips related to oxidation were detected, within our experimental accuracy.

Next, we analyzed the GGST/ST core-shell NWs with different shell diameters. Figure 3c shows the XPS results on the NWs having 30 nm of shell thickness. The spectrum demonstrates the presence of Sb and Te elements only, with the peaks located at 32.70 ± 0.1 eV (Sb_4d) and 40.15 ± 0.1 eV (Te_4d), respectively. No presence of Ge was detected. This could be due to the fact that the ultimate depth that our XPS system could get data from is about 6 nm, this being a plausible reason for not observing the core contribution. Thus, in order to have a complete characterization of the core-shell structure and validate our results from an interdiffusion point of view, a lower shell thickness is required to probe the interface between the GGST and the ST shell. Moreover, it could be interesting to see whether Ge still remained unaffected as a core material after the shell’s growth. Thus, we investigated the GGST/ST NWs with a conformal shell of 10 nm; Figure 3d displays the obtained XPS measurements. Only the presence of Sb and Te was detected, with peaks positions at 32.9 ± 0.1 eV (Sb_4d) and 40.2 ± 0.1 eV (Te_4d), respectively. Here, the absence of the Ge peak means that the core-shell interface is sharp and no Ge diffusion towards the shell takes place. A possible reason can be the room temperature shell deposition, that prevents interdiffusion. Thus, from the obtained results, we could confirm that the GGST core NWs are completely covered with the ST shell, and that Ge does not diffuse into the shell.

It should anyway be considered that all the surfaces of substrate and NWs are covered with Sb_2_Te_3_, whereas Ge falls only below the NWs’ shell surface, and there is a low density of NWs compared to the substrate surface area. This makes the Sb and Te signals naturally larger than the Ge one, because they come from a wider region. Even when the few NWs are looked at, the Ge signal is attenuated by the shell thickness. The Sb_4d peak buries the Ge_3d (IMFP (λ), for Ge_3d is ~2 nm [35] in this energy region, so that the latter is hardly or not recognizable in the core-shell samples. For this reason, we considered a different energy region in which to study the Ge signal without any disturbance from other elements in the sample, such as Te and Sb. As mentioned before, (IMFP (λ) for Ge_2p is ~0.9 nm [36]. Thus, it is a suitable peak for looking at the Ge signal in core-shell NWs with 10 nm shell, as being isolated from other relevant peaks of ST.

Figure 4 shows the XPS spectra for Ge_2p_3/2_ region of GGST core NWs and GGST/ST core-shell NWs with 10 nm shell thickness. The dots in the figures are the raw data, and the solid lines are the fitted data. The gray color shows the inelastic background, fitted by the Shirley method, and the individual deconvoluted peaks used for fitting. Thus, we were able to detect the Ge_2p peak in core-shell NWs with 10 nm shell thickness. Upon comparing such results with Figure 3b, an increase in the binding energy of the Ge_2p peak on the core-shell NWs was observed.

Moreover, looking at the increase in the binding energy of the Ge_2p peak, we concluded that the Ge atoms on the surface of the core GGST NWs tended to bond chemically with the Sb atoms from the shell, as the Ge-Sb bonding energy is more stable and has a low equilibrium potential. These results validated the existence of the chemical interaction between the GGST core and the ST shell. We noted that the same interaction appeared in the GT/ST case (data not shown).

**Figure 5:**
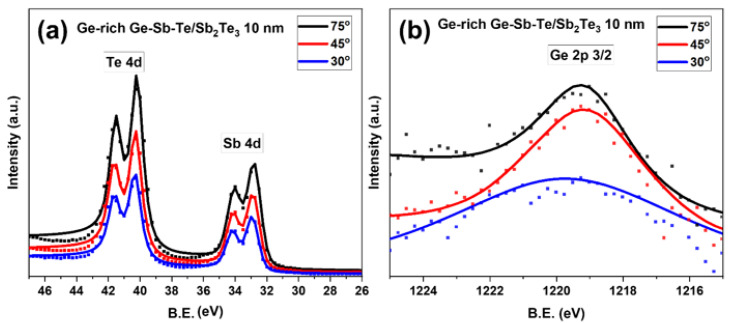
XPS spectra for (**a**) Te_4d, Sb_4d; and (**b**) Ge_2p regions collected at 30° (blue), 45° (red) and 75° (black) take-off angles for GGST/ST core-shell NWs with 10 nm shell thickness; (dots– experimental data; solid lines—fit).

We further analyzed the GGST/ST core-shell NWs having the shell thickness of 10 nm with different photoelectrons take-off angles. Figure 5a displays the fitted XPS spectra, with the same procedure as in Figure 3, featured by peaks at 32.70 ± 0.1 eV and 40.15 ± 0.1 eV, for Sb_4d and Te_4d, respectively. The increase in the peak intensity with the same peak position as a function of the incident angle was observed. However, the Ge peak was not observed. As discussed above, the very high peak intensity from the Te and Sb signals, which are related to the Sb_2_Te_3_ conformal coating, would overcome the Ge peak generated by the core NWs, having much less intensity. So, the Ge_2p peak at a higher binding energy was considered. Figure 5b shows the fitted XPS measurements of Ge_2p repeated with three different incident take-off angles, namely 30, 45, and 75 degrees. The Ge_2p core peak was observed for all angles at 1219.3 ± 0.1 eV (Ge_2p_3/2_). By comparing the obtained results from different incident angles, an increase in peak intensity with the angle was observed, and it could demonstrate the existence of GGST as the core material in the core-shell NWs.

Next, upon comparing the position of Ge_2p_3/2_ in GGST/ST core-shell NWs (1219.3 eV) and GGST NWs (1218.3 eV, Figure 3b), a chemical shift in the binding energy of 1 eV was observed. Such a shift could be due to the chemical bond of GGST with ST.

**Figure 6:**
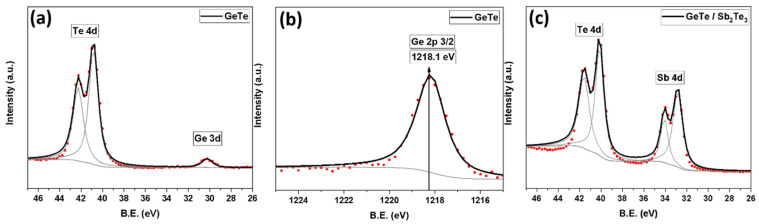
XPS spectra for (**a**) Ge_3d and Te_4d; (**b**) Ge_2p region for GT core NWs; (**c**) Sb_4d and Te_4d region for GT/ST core-shell with 10 nm shell thickness; (dots–experimental data; solid lines—fit).

Further, we performed XPS measurements on the GT core and GT/ST core-shell NWs. Figure 6a,b shows a clear and sharp presence of Ge_3d, Te_4d and Ge_2p_3/2_ core peaks originating from the GT core NWs. The dots in the figures are the raw data, and the solid lines are the fitted data. The gray color shows the inelastic background, fitted by the Shirley method, and the individual deconvoluted peaks used for fitting. The same data representation and analysis apply to Figure 6c. Figure 6c reports the XPS analysis from the GT core NWs coated at room temperature with a 10 nm thick shell of ST. The spectrum shows clear Sb_4d and Te_4d peaks and an absence of the Ge_3d peak, suggesting the uniform coating of the ST shell over the GT core NWs. In addition, the Te_4d and Sb_4d peaks have exactly the same energy as in the GGST core-shell NWs with ST as the shell. It demonstrates that the shell has preserved its properties independently of the core material. The XPS analysis over the Ge_2p_3/2_ peak was also acquired (see Supplementary Materials, Figure S1); the peak binding energy at 1218.5 eV is slightly increased in comparison with the value from the GT core NWs (1218.1 eV, Figure 6b), possibly claiming for the existence of a chemical interaction between the core and shell also for the GT/ST NWs.

Further, it is worth noting that the binding energy of Ge 2p_3/2_ in GGST (1218.3 ± 0.1 eV) matches within the error bar, the value in GT (1218.1 ± 0.1 eV) meanwhile moves to higher energy in Ge_2_Sb_2_Te_3_ (1219.5 ± 0.1 eV), as reported in the literature [37]. Therefore, the chemical bonding of Ge in GGST is more similar to the Ge in GT than to the Ge in Ge_2_Sb_2_Te_5_.

However, after the growth of the shell, the Ge_2p peak binding energy is 1219.3 ± 0.1 eV in the GGST, being close to the value of 1219 ± 0.1 eV found in Ge_2_Sb_2_Te_5_ and 1218.4 ± 0.1 eV in the GT cases. Such an observed trend may be related to the Ge atoms from the core NWs bonded with Sb atoms from the shell of NWs at the core–shell interface, supported by the tendency to form Ge–Sb bonding to reduce the overall potential energy of the alloy. When the value of Ge–Sb chemical bonding increases, the potential energy of the Ge atom decreases, however a detailed investigation on this mechanism is beyond the scope of this paper and would deserve more investigation.

## 4. Conclusions

In summary, Ge-rich Ge-Sb-Te and GeTe core NWs with a Sb_2_Te_3_ shell with thickness down to 10 nm were synthesized via MOCVD. The morphology showed a continuous shell coating all over the core NWs. The XRD analysis revealed that the core structure of the NWs was not altered by the shell deposition. The XPS measurements gave insight into the interaction between the NWs core and shell, with an indication about the Ge chemical state at the interface. The chemical shift of the Ge_2p peak was observed, confirming the interaction of the core and the shell. Angular-resolved XPS spectra indicated the absence of interdiffusion between the core and shell elements, suggesting that their structural phase can change independently, based on the alloy composition. This work demonstrated a straightforward method to provide efficient core-shell NW heterostructures, formed by two-phase change materials having different crystallization temperatures and reversible switching speed. This is particularly useful for comparison with corresponding planar multilayered PCM cells. Our results could be helpful in the fundamental understanding of phase change materials for the realization of memory devices and, in particular, for a comparison with corresponding planar multilayered PCM cells.

## Data Availability

The data that support the findings of this study are available from the corresponding authors upon reasonable request.

## Supplementary Materials

The following supporting information can be downloaded at: https://www.mdpi.com/article/10.3390/nano12101623/s1, Table S1: Samples analyzed and their corresponding experimental XPS peak positions and FWHM; Figure S1: XPS spectra of Ge_2p region for GT/ST core-shell with 10 nm shell thickness; (dots–experimental data, solid lines–fit).

## References

[1] Ovshinsky S.R. (1968). Reversible Electrical Switching Phenomena in Disordered Structures. Phys. Rev. Lett..

[2] Lankhorst M.H.R., Ketelaars B.W.S.M.M., Wolters R.A.M. (2005). Low-cost and nanoscale non-volatile memory concept for future silicon chips. Nat. Mater..

[3] Liu Z., Xu J., Chen D., Shen G. (2014). Flexible electronics based on inorganic nanowires. Chem. Soc. Rev..

[4] Meyyappan M., Lee J.S. (2010). The quiet revolution of inorganic nanowires. IEEE Nanotechnol. Mag..

[5] Eggleton B.J., Luther-Davies B., Richardson K. (2011). Chalcogenide photonics. Nat. Photonics.

[6] Yu B., Sun X., Ju S., Janes D.B., Meyyappan M. (2008). Chalcogenide-nanowire-based phase change memory. IEEE Trans. Nanotechnol..

[7] Yamada N., Ohno E., Nishiuchi K., Akahira N., Takao M. (1998). Rapid-phase transitions of GeTe-Sb_2_Te_3_ pseudobinary amorphous thin films for an optical disk memory. J. Appl. Phys..

[8] Nishi Y. (2014). Advances in Non-Volatile Memory and Storage Technology.

[9] Lee S.H., Ko D.K., Jung Y., Agarwal R. (2006). Size-dependent phase transition memory switching behavior and low writing currents in GeTe nanowires. Appl. Phys. Lett..

[10] Rodgers P., Heath J. (2009). Nanoscience and Technology: A Collection of Reviews from Nature Journals.

[11] Cecchini R., Gajjela R.S.R., Martella C., Wiemer C., Lamperti A., Nasi L., Lazzarini L., Nobili L.G., Longo M., Cecchini R. (2019). High-Density Sb2Te3 Nanopillars Arrays by Templated, Bottom-Up MOCVD Growth. Small.

[12] Cecchini R., Selmo S., Wiemer C., Fanciulli M., Rotunno E., Lazzarini L., Rigato M., Pogany D., Lugstein A., Longo M. (2019). In-doped Sb nanowires grown by MOCVD for high speed phase change memories. Micro Nano Eng..

[13] Cecchini R., Selmo S., Wiemer C., Rotunno E., Lazzarini L., De Luca M., Zardo I., Longo M. (2017). Single-step Au-catalysed synthesis and microstructural characterization of core–shell Ge/In–Te nanowires by MOCVD. Mater. Res. Lett..

[14] Selmo S., Cecchini R., Cecchi S., Wiemer C., Fanciulli M., Rotunno E., Lazzarini L., Rigato M., Pogany D., Lugstein A. (2016). Low power phase change memory switching of ultra-thin In3Sb1Te2 nanowires. Appl. Phys. Lett..

[15] Longo M. (2019). Advances in nanowire PCM. Advances in Non-Volatile Memory and Storage Technology.

[16] Wu Y., Fan R., Yang P. (2002). Block-by-Block Growth of Single-Crystalline Si/SiGe Superlattice Nanowires. Nano Lett..

[17] Björk M.T., Ohlsson B.J., Sass T., Persson A.I., Thelander C., Magnusson M.H., Deppert K., Wallenberg L.R., Samuelson L. (2002). One-dimensional heterostructures in semiconductor nanowhiskers. Appl. Phys. Lett..

[18] Gudiksen M.S., Lauhon L.J., Wang J., Smith D.C., Lieber C.M. (2002). Growth of nanowire superlattice structures for nanoscale photonics and electronics. Nature.

[19] Lauhon L.J., Gudlksen M.S., Wang D., Lieber C.M. (2002). Epitaxial core-shell and core-multishell nanowire heterostructures. Nature.

[20] Dong Y., Yu G., McAlpine M.C., Lu W., Lieber C.M. (2008). Si/a-Si core/shell nanowires as nonvolatile crossbar switches. Nano Lett..

[21] Jung Y., Ko D.K., Agarwal R. (2007). Synthesis and structural characterization of single-crystalline branched nanowire heterostructures. Nano Lett..

[22] Yu D., Wu J., Gu Q., Park H. (2006). Germanium telluride nanowires and nanohelices with memory-switching behavior. J. Am. Chem. Soc..

[23] Meister S., Peng H., McIlwrath K., Jarausch K., Zhang X.F., Cui Y. (2006). Synthesis and characterization of phase-change nanowires. Nano Lett..

[24] Nukala P., Lin C.C., Composto R., Agarwal R. (2016). Ultralow-power switching via defect engineering in germanium telluride phase-change memory devices. Nat. Commun..

[25] Longo M., Wiemer C., Salicio O., Fanciulli M., Lazzarini L., Rotunno E. (2011). Au-catalyzed self assembly of GeTe nanowires by MOCVD. J. Cryst. Growth.

[26] Lee S.H., Jung Y., Agarwal R. (2007). Highly scalable non-volatile and ultra-low-power phase-change nanowire memory. Nat. Nanotechnol..

[27] Longo M., Stoycheva T., Fallica R., Wiemer C., Lazzarini L., Rotunno E. (2013). Au-catalyzed synthesis and characterisation of phase change Ge-doped Sb-Te nanowires by MOCVD. J. Cryst. Growth.

[28] Jung Y., Lee S.H., Ko D.K., Agarwal R. (2006). Synthesis and characterization of Ge_2_Sb_2_Te_5_ nanowires with memory switching effect. J. Am. Chem. Soc..

[29] Jung Y., Lee S.H., Jennings A.T., Agarwal R. (2008). Core-shell heterostructured phase change nanowire multistate memory. Nano Lett..

[30] Kumar A., Cecchini R., Wiemer C., Mussi V., De Simone S., Calarco R., Scuderi M., Nicotra G., Longo M. (2021). Phase Change Ge-Rich Ge–Sb–Te/Sb_2_Te_3_ Core-Shell Nanowires by Metal Organic Chemical Vapor Deposition. Nanomaterials.

[31] Kumar A., Cecchini R., Wiemer C., Mussi V., De Simone S., Calarco R., Scuderi M., Nicotra G., Longo M. (2021). MOCVD Growth of GeTe/Sb_2_Te_3_ Core–Shell Nanowires. Coatings.

[32] http://maud.radiographema.eu/.

[33] Egerton R.F. (1996). Electron Energy-Loss Spectroscopy in the Electron Microscope.

[34] Canvel Y., Lagrasta S., Boixaderas C., Barnola S., Mazel Y., Martinez E. (2019). Study of Ge-rich GeSbTe etching process with different halogen plasmas. J. Vac. Sci. Technol. A.

[35] Shinotsuka H., Tanuma S., Powell C.J., Penn D.R. (2015). Calculations of electron inelastic mean free paths. X. Data for 41 elemental solids over the 50 eV to 200 keV range with the relativistic full Penn algorithm. Surf. Interface Anal..

[36] Wang W., Lei D., Dong Y., Gong X., Tok E.S., Yeo Y.C. (2017). Digital Etch Technique for Forming Ultra-Scaled Germanium-Tin (Ge_1−x_Sn_x_) Fin Structure. Sci. Rep..

[37] Song K.H., Baek S.C., Lee H.Y. (2012). Amorphous-to-crystalline phase transformation in (GeTe)_x_(Sb_2_Te_3_) (x = 0.5, 1, 2, 8) thin films. J. Korean Phys. Soc..

