# Supplementary material for: Interface Analysis of MOCVD Grown GeTe/Sb2Te3 and Ge-Rich Ge-Sb-Te/Sb2Te3 Core-Shell Nanowires
Source: Nanomaterials (Basel). 2022 May 10;12(10):1623. doi: 10.3390/nano12101623 (PMC9146787; doi:10.3390/nano12101623)
Supplement: Supplementary file 1 [file nanomaterials-12-01623-s001.zip › nanomaterials-1693739-supplementary.pdf]

Supplementary Materials

Interface Analysis of MOCVD Grown GeTe/Sb_2Te_3 and Ge-rich Ge-Sb-Te/Sb_2Te_3 Core-shell Nanowires

Arun Kumar [1,2,*], Seyed Ariana Mirshokraee [1,3], Alessio Lamperti [1], Matteo Cantoni [3], Massimo Longo [4,*] and Claudia Wiemer [1]

[1] CNR—Institute for Microelectronics and Microsystems, Via C. Olivetti 2, 20864 Agrate Brianza, Italy; s.mirshokraee@campus.unimib.it (S.A.M.); alessio.lamperti@mdm.imm.cnr.it (A.L.); claudia.wiemer@mdm.imm.cnr.it (C.W.)
[2] Department of Physics 'E.R. Caianiello', University of Salerno, Via G. Paollo I 132, 84084 Salerno, Italy
[3] Department of Physics, Politecnico di Milano, Via G. Colombo 81, 20133 Milano, Italy; matteo.cantoni@polimi.it
[4] CNR—Institute for Microelectronics and Microsystems, Via del Fosso del Cavaliere 100, 00133 Rome, Italy
* Correspondence: arun.kumar@mdm.imm.cnr.it (A.K.); massimo.longo@artov.imm.cnr.it (M.L.)

Table S1. Samples analyzed and their corresponding experimental XPS peak positions and FWHM.

Sample	Peaks	Position (eV)	FWHM (eV)
Ge-rich Ge-Sb-Te	Te 4d 3/2	40.320 ±0.1	1.034
	Te 4d 1/2	41.910 ±0.1	0.894
	Sb 4d 3/2	32.870 ±0.1	0.550
	Sb 4d 1/2	34.100 ±0.1	0.935
	Ge 3d	30.249 ±0.1	1.193
	Ge 2p 3/2	1218.300 ±0.1	1.541
Ge-rich Ge-Sb-Te/ Sb_2Te_3	Te 4d 3/2	40.270 ±0.1	1.090
	Te 4d 1/2	41.650 ±0.1	0.960
	Sb 4d 3/2	32.920 ±0.1	1.050
	Sb 4d 1/2	34.150 ±0.1	0.700
	Ge 2p 3/2	1219.300 ±0.1	3.540
GeTe	Te 4d 3/2	40.84 ±0.1	1.020
	Te 4d 1/2	42.270 ±0.1	1.150
	Ge 3d	30.235 ±0.1	1.140
	Ge 2p 3/2	1218.188 ±0.1	1.519
GeTe/ Sb_2Te_3	Te 4d 3/2	40.140 ±0.1	0.930
	Te 4d 1/2	41.590 ±0.1	1.180
	Sb 4d 3/2	32.840 ±0.1	1.070
	Sb 4d 1/2	34.090 ±0.1	0.830
	Ge 2p 3/2	1218.430±0.1	2.060

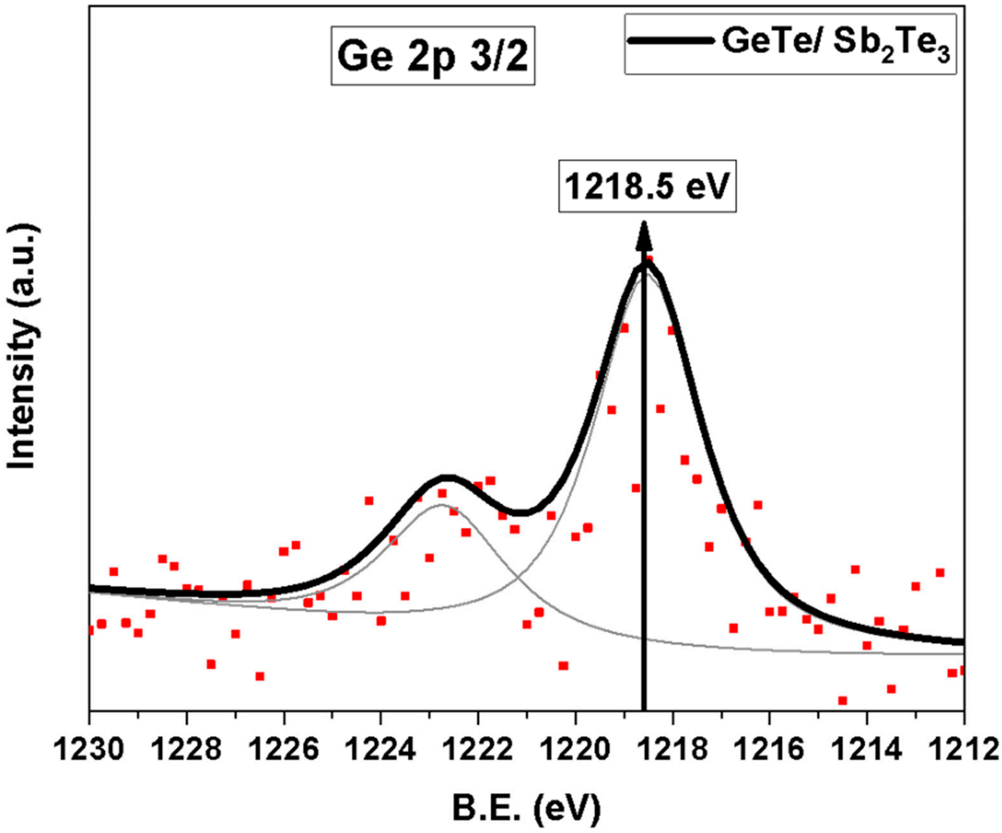

Figure S1. XPS spectra of Ge_2p region for GT/ST core-shell with 10 nm shell thickness; (dots – experimental data, solid lines – fit).
